# Effect of granulocyte colony-stimulating factor on toxicities after CAR T cell therapy for lymphoma and myeloma

**DOI:** 10.1038/s41408-022-00741-2

**Published:** 2022-11-01

**Authors:** Kevin Charles Miller, Patrick Connor Johnson, Jeremy S. Abramson, Jacob D. Soumerai, Andrew J. Yee, Andrew R. Branagan, Elizabeth K. O’Donnell, Anna Saucier, Caron A. Jacobson, Matthew J. Frigault, Noopur S. Raje

**Affiliations:** 1 Department of Medicine, Massachusetts General Hospital, Boston, MA USA; 2 Center for Lymphoma, Massachusetts General Hospital, Boston, MA USA; 3 Cellular Immunotherapy Program, Massachusetts General Hospital, Boston, MA USA; 4 Center for Multiple Myeloma, Massachusetts General Hospital, Boston, MA USA; 5 Tufts University School of Medicine, Boston, MA USA; 6 Immune Effector Cell Therapy Program, Dana-Farber Cancer Institute, Boston, MA USA; 7 Blood and Marrow Transplant Program, Massachusetts General Hospital, Boston, MA USA

**Keywords:** Immunotherapy, Lymphoma, Myeloma

## Abstract

Chimeric antigen receptor T cells (CAR T) are groundbreaking therapies but may cause significant toxicities including cytokine release syndrome (CRS), immune effector cell-associated neurotoxicity syndrome (ICANS), and cytopenias. Granulocyte colony-stimulating factor (G-CSF) is often used to mitigate neutropenia after CAR T, but there is no consensus recommended strategy due to hypothesized, but largely unknown risks of exacerbating toxicities. To investigate the impact of G-CSF, we retrospectively analyzed 197 patients treated with anti-CD19 CAR T for lymphoma and 47 patients treated with anti-BCMA CAR T for multiple myeloma. In lymphoma, 140 patients (71%) received prophylactic G-CSF before CAR T (mostly pegylated G-CSF) and were compared with 57 patients (29%) treated with G-CSF after CAR T or not exposed. Prophylactic G-CSF was associated with faster neutrophil recovery (3 vs. 4 days, *P* < 0.01) but did not reduce recurrent neutropenia later. Prophylactic G-CSF was associated with increased grade ≥2 CRS (HR 2.15, 95% CI 1.11–4.18, *P* = 0.02), but not ICANS. In multiple myeloma, prophylactic G-CSF was not used; patients were stratified by early G-CSF exposure (≤2 days vs. ≥3 days after CAR T or no exposure), with no significant difference in toxicities. Future trials should clarify the optimal G-CSF strategy to improve outcomes after CAR T.

## Introduction

Chimeric antigen receptor T cell therapy (CAR T) has made a transformative impact on the treatment of blood cancers including lymphoma and multiple myeloma [1–5]. CAR T cells can persist in vivo for over a decade and offer the possibility of deep responses in highly refractory patients [6]. Nevertheless, the promise of CAR T is tempered by immune toxicities, namely cytokine release syndrome (CRS) and immune effector cell-associated neurotoxicity syndrome (ICANS), which may cause significant morbidity and rarely treatment-related mortality [7–9]. Further, although most patients experience transient cytopenias immediately after CAR T, prolonged hematologic toxicity lasting months occurs in a sizable fraction of patients [10–13]. Contributors toward hematologic toxicity include lymphodepleting chemotherapy (usually fludarabine and cyclophosphamide), decreased baseline hematopoietic reserve, and the inflammatory sequalae of CAR T cell activity in the bone marrow microenvironment [13].

Granulocyte colony-stimulating factor (G-CSF) is often used prophylactically to expedite neutrophil recovery in patients treated with chemotherapy, but there is no consensus on the optimal use of G-CSF for patients receiving CAR T [14–16]. Part of the uncertainty stems from emerging understanding of the pathophysiology of CRS and ICANS, which each involve the activation of recipient myeloid cells and upregulation of myeloid-derived cytokines [1, 17–21]. Further, high serum G-CSF and GM-CSF levels have been associated with severe ICANS, and GM-CSF blockade has been shown to reduce cytokine release and neuroinflammation in a xenograft CAR T model [4, 17, 19, 20, 22]. Thus, due to concerns about stimulating myeloid progenitor cells and potentially exacerbating the severity of CRS and ICANS, there has been justifiable hesitancy amongst the cell therapy community to recommend a strategy for using G-CSF to counteract post-CAR T neutropenia [15, 23, 24]. However, few studies have investigated the effect of G-CSF administration on CAR T toxicities to date [25–27].

Notably, the package insert for tisagenlecleucel recommends avoiding growth factors until three weeks after CAR T or until CRS has resolved; the package inserts for axicabtagene ciloleucel and idecabtagene vicleucel do not specifically comment [28–30]. Most institutions have created their own policy around G-CSF utilization in CAR T patients [15, 31]. Our centers have sometimes used pegylated G-CSF prior to CAR T, which has clinical effects roughly equivalent to eleven consecutive daily injections of G-CSF [32]. The outcomes of a prophylactic G-CSF approach compared to other strategies, such as administering G-CSF after CAR T to treat neutropenia, or avoidance of G-CSF altogether, are unknown. Given the lack of clarity on how G-CSF affects the risks of CRS, ICANS, cytopenias, and infections after CAR T, we retrospectively interrogated cohorts of patients treated with CAR T for lymphoma and multiple myeloma at our centers. We intend these data to generate hypotheses for prospective trials with different G-CSF implementation guidelines to improve the safety of CAR T.

## Methods

This study was approved by our Institutional Review Board and conducted in accordance with the Declaration of Helsinki. The primary cohort included adults ≥18 years old treated with commercial anti-CD19 CAR T cells (tisagenlecleucel or axicabtagene ciloleucel) for lymphoma at Massachusetts General Hospital and Brigham and Women’s Hospital/Dana-Farber Cancer Institute after their respective United States Food and Drug Administration approval dates, from October 18, 2017 to December 31, 2019. The following diagnoses were eligible: relapsed/refractory diffuse large B-cell lymphoma (DLBCL), including DLBCL transformed from low-grade lymphoma, high-grade B-cell lymphoma (HGBCL), follicular lymphoma (FL), primary mediastinal B-cell lymphoma (PMBCL), mantle cell lymphoma, and marginal zone lymphoma. Patients who received novel adjuvant therapies after CAR T as part of clinical trials, including PD-1 inhibitors and ibrutinib, were excluded. Lymphoma response and disease progression were defined according to the International Working Group criteria [33]. A second cohort was collected and analyzed including adults ≥18 years old treated with investigational anti-BCMA CAR T cells for relapsed/refractory multiple myeloma at Massachusetts General Hospital from November 1, 2016 to September 1, 2021. The International Myeloma Working Group criteria were used to assess treatment response [34]. CRS and ICANS were re-graded based on review of the medical record according to American Society for Transplantation and Cellular Therapy (ASTCT) consensus recommendations [35]. CRS grading was based on documentation of temperature, blood pressure, oxygenation, crystalloid fluid administration, and vasopressor use. ICANS grading was based on documentation of immune effector cell encephalopathy (ICE) scores, level of consciousness, seizures and neuroimaging findings. Mild, moderate, and severe neutropenia were defined as absolute neutrophil count (ANC) < 1.5 × 10^9^/L, < 1.0 × 10^9^/L, and < 0.5 × 10^9^/L, respectively. Neutrophil recovery was defined as ANC > 0.5 × 10^9^/L for three consecutive days. Severe thrombocytopenia was defined as platelets < 20 × 10^9^/L; engraftment was defined as platelets > 20 × 10^9^/L for three consecutive days, with absence of transfusions for seven days.

The Mann–Whitney U test was used to compare continuous variables. Fisher’s exact test was used to compare categorical variables. Progression-free survival (PFS) was defined as the time from CAR T cell infusion to either disease progression or death. Overall survival (OS) was defined as the time from CAR T cell infusion to death. The probability of PFS and OS were estimated using the Kaplan-Meier method. Patients who remained alive were censored at the time of last follow-up. PFS and OS were compared using the log-rank test. The time of last follow-up for both cohorts was January 1, 2022. Median follow-up time was calculated using the reverse Kaplan-Meier method [36].

The cumulative incidence of CRS and ICANS were estimated from the date of CAR T cell infusion and compared using the method of Gray, with patients censored at the time of last follow-up or death [37]. In addition, to investigate the effect of G-CSF on worsening of low-grade CRS in the lymphoma cohort, the cumulative incidence of grade ≥2 CRS from the onset of grade 1 CRS was estimated. The cumulative incidence of time to neutrophil recovery was summarized from the time of severe neutropenia, with patients censored at the time of last follow-up or death if recovery did not occur prior. Finally, a competing risk model was generated for the endpoint of either recurrent severe neutropenia or treatment with G-CSF after day ten post-CAR T infusion, with disease progression and death as competing events.

Cox proportional hazards models were developed for time to grade ≥2 CRS, grade ≥2 ICANS and neutrophil recovery in the lymphoma cohort using a forward selection method. Logistic regression models were developed for severe neutropenia and thrombocytopenia. Univariate models included age, sex, CAR T construct, Eastern Cooperative Oncology Group (ECOG) performance status, lines of therapy prior to CAR T, receipt of bridging chemotherapy, time from diagnosis to CAR T, pre-lymphodepletion ANC, hemoglobin, platelets, lactate dehydrogenase (LDH) and albumin. Variables with *P* < 0.10 (Wald test) in univariate analyses were included in multivariate models, in addition to the variable of interest (prophylactic G-CSF) if not already included from univariate analyses. Hazard ratios (HR) and odds ratios (OR) were shown with 95% confidence intervals (CI). For the competing risk model, subdistribution HR were generated using the method of Fine and Gray [38]. Statistical significance was denoted as *P* < 0.05. Analyses and figures were generated using R version 3.6.1 and R studio version 2022.02.2 + 485.

## Results

### Characteristics of patients treated with anti-CD19 CAR T for lymphoma

We identified 197 patients who received anti-CD19 CAR T cells for the treatment of relapsed/refractory lymphoma. Most patients (*N* = 166, 84%) were treated with axicabtagene ciloleucel; the other 31 (16%) received tisagenlecleucel. The median age at CAR T was 62. The diagnoses included DLBCL (*N* = 95, 48%), transformed DLBCL (*N* = 40, 20%), HGBCL (*N* = 28, 14%), FL (*N* = 19, 10%), PMBCL (*N* = 10, 5%) and mantle cell or marginal zone lymphoma (*N* = 5, 3%).

Patients received a median of three prior lines of therapy (range 1–9). Bridging therapy was administered in 77 patients (39%) prior to CAR T. Fifty-four patients (27%) underwent prior autologous hematopoietic cell transplantation (HCT). Five patients (3%) received prior allogeneic HCT. All received fludarabine and cyclophosphamide for lymphodepletion per standard protocols. Most patients (*N* = 177, 90%) received antibiotic prophylaxis, chiefly with fluoroquinolones (*N* = 156, 79%); triazole fungal prophylaxis was less frequent (*N* = 20, 10%). The median follow-up time was 29.9 months (95% CI 28.3–32.0).

### Characteristics of G-CSF given before and after anti-CD19 CAR T for lymphoma

Initiation of G-CSF before or after CAR T was determined contemporaneously by treating physicians. To avoid confounding based on the clinical course after CAR T cell infusion [10, 12, 13], we stratified patients by exposure to prophylactic G-CSF prior to CAR T. There were 140 patients (71%) exposed to prophylactic G-CSF. Most of these, 126 of 140, were treated with pegylated G-CSF, starting a median of two days prior to CAR T. The other 14 of 140 patients received daily G-CSF injections starting a median of two days prior to CAR T, for a median of nine consecutive doses (range, 6–28).

On the other hand, 57 patients (29%) were not exposed to G-CSF immediately prior to CAR T, and hereafter referred to as the control group. Forty-two of the 57 control group patients went on to receive G-CSF after CAR T to treat neutropenia, a median of six days post-infusion (range, 1–23). The other 15 of 57 did not receive any G-CSF within 30 days after CAR T. Baseline characteristics are shown in Table 1. The prophylactic G-CSF and control groups were generally comparable, except bridging chemotherapy and tisagenlecleucel were more commonly used in the control group.

**Table 1:** Baseline characteristics in lymphoma cohort.

	Prophylactic G-CSF^a^ (*N* = 140)	Control^b^ (*N* = 57)	Total (*N* = 197)
*Age at CAR T cell Infusion*			
Median [Min, Max]	62 [19, 80]	62 [21, 82]	62 [19, 82]
*Sex*			
Female	53 (37.9%)	23 (40.4%)	76 (38.6%)
*Disease subtype*			
DLBCL	68 (48.6%)	27 (47.4%)	95 (48.2%)
Transformed DLBCL	22 (15.7%)	18 (31.6%)	40 (20.3%)
High-grade B-cell lymphoma	22 (15.7%)	6 (10.5%)	28 (14.2%)
Follicular lymphoma	18 (12.9%)	1 (1.8%)	19 (9.6%)
Primary mediastinal B-cell lymphoma	5 (3.6%)	5 (8.8%)	10 (5.1%)
Other (mantle cell and marginal zone lymphoma)	5 (3.6%)	0 (0%)	5 (2.5%)
*Number of prior lines of therapy*			
1–2	47 (33.6%)	20 (35.1%)	67 (34.0%)
3	37 (26.4%)	18 (31.6%)	55 (27.9%)
4	28 (20.0%)	10 (17.5%)	38 (19.3%)
≥5	28 (20.0%)	9 (15.8%)	37 (18.8%)
*Bridging therapy prior to CAR T*			
Yes	39 (27.9%)	38 (66.7%)	77 (39.1%)
*Prior autologous transplant*			
Yes	39 (27.9%)	15 (26.3%)	54 (27.4%)
*Prior allogeneic transplant*			
Yes	4 (2.9%)	1 (1.8%)	5 (2.5%)
*ECOG performance status at CAR T*			
0	66 (47.1%)	29 (50.9%)	95 (48.2%)
1	65 (46.4%)	25 (43.9%)	90 (45.7%)
≥2	9 (6.4%)	3 (5.3%)	12 (6.1%)
*CAR T cell construct*			
Axicabtagene ciloleucel	134 (95.7%)	32 (56.1%)	166 (84.3%)
Tisagenlecleucel	6 (4.3%)	25 (43.9%)	31 (15.7%)
*Time from diagnosis to CAR T (months)*			
Median [Min, Max]	22.8 [1.3, 415]	15.8 [2.5, 346]	20.9 [1.3, 415]
*White blood cell count (*×*10*^*9*^*/L)*^c^			
Median [Min, Max]	5.47 [0.01, 45.8]	5.70 [1.1, 13.9]	5.55 [0.01, 45.8]
*Absolute neutrophil count (*×*10*^*9*^*/L)*^c^			
Median [Min, Max]	4.0 [0.01, 20.8]	4.0 [0.4, 11.4]	4.0 [0.01, 20.8]
*Hemoglobin (g/dL)*^c^			
Median [Min, Max]	11.7 [7.6, 15.4]	11.3 [7.3, 14.7]	11.5 [7.3, 15.4]
*Platelet Count (*×*10*^*9*^*/L)*^c^			
Median [Min, Max]	175 [23, 599]	168 [15, 360]	174 [15, 599]
*Lactate Dehydrogenase (IU/L)*^c^			
Median [Min, Max]	211 [85, 1810]	254 [116, 945]	215 [85, 1810]
*Albumin (g/dL)*^c^			
Median [Min, Max]	4.0 [2.1, 5.0]	4.0 [2.3, 4.7]	4.0 [2.1, 5.0]

^a^Prophylactic G-CSF refers to exposure to G-CSF prior to CAR T cell infusion.

^b^Control group includes patients who either received G-CSF after CAR T cell infusion (*N* = 42) or were not exposed to G-CSF (*N* = 15).

^c^Most recent values measured immediately prior to initiation of lymphodepletion chemotherapy.

### Neutropenia after anti-CD19 CAR T for lymphoma

Most patients (*N* = 187, 95%) developed mild neutropenia (ANC < 1.5 × 10^9^/L) immediately after CAR T. Further, 151 patients (77%) developed severe neutropenia (ANC < 0.5 × 10^9^/L), occurring within a median of three days (range, 0–11). A multivariate logistic regression model was generated, whereby CAR T construct (axicabtagene ciloleucel) was associated with an increased relative odds of severe neutropenia (OR 5.96, 95% CI 1.90–21.20, *P* < 0.01). There was a trend toward prophylactic G-CSF being associated with a decreased relative odds of severe neutropenia in a multivariate analysis (OR 0.33, 95% CI 0.10–0.93, *P* = 0.05)(Supplemental Table 1).

**Fig. 1:**
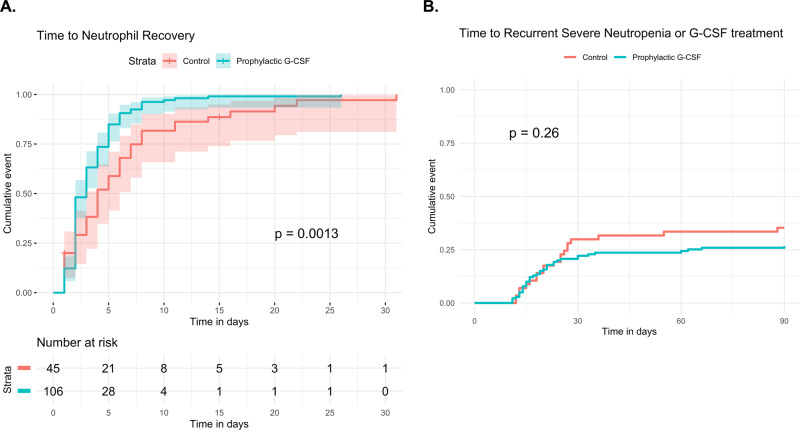
Neutropenia in lymphoma patients treated with anti-CD19 CAR T cells. **A** Time to neutrophil recovery in the 151 lymphoma patients treated with anti-CD19 CAR T cells who developed severe neutropenia. Patients were stratified by whether they received prophylactic G-CSF (*N* = 106) or not (control, *N* = 45). Median time to neutrophil recovery was significantly shorter in the prophylactic G-CSF group (3 vs. 4 days, *P* < 0.01). In a multivariate Cox proportional hazards model, administration of prophylactic G-CSF prior to CAR T retained significance for faster neutrophil recovery (HR 2.11, 95% CI 1.39–3.20, *P* < 0.01). **B** Among 197 lymphoma patients treated with anti-CD19 CAR T cells, the cumulative incidence of either later severe neutropenia or treatment with G-CSF after day 10 post-CAR T infusion is shown, with progression or death as a competing risk (not shown). Prophylactic G-CSF did not significantly reduce the risk of recurrent severe neutropenia or later G-CSF treatment (Fine-Gray, HR 0.74, 95% CI 0.43–1.25, *P* = 0.26). 95% confidence intervals are represented by shading.

The median duration of severe neutropenia was three days (range, 1–37). Time to neutrophil recovery (ANC > 0.5 × 10^9^/L) was faster in the prophylactic G-CSF group compared to the control group (median 3 vs. 4 days, *P* < 0.01) (Fig. 1A). Median time to ANC > 1.0 × 10^9^/L was also faster with prophylactic G-CSF (median 3 vs. 5 days, *P* = 0.01). In a univariate Cox regression model, predictors of neutrophil recovery (ANC > 0.5 × 10^9^/L) included bridging chemotherapy, hemoglobin, platelets, albumin, LDH, and prophylactic G-CSF (Table 2). Prophylactic G-CSF retained significance for faster neutrophil recovery in a multivariate analysis (HR 2.11, 95% CI 1.39–3.20, *P* < 0.01).

**Table 2:** Cox proportional hazards model for time to neutrophil recovery in lymphoma cohort.

Variable	Univariate analysis^a^				Multivariate analysis			
	HR	Lower 95% CI	Upper 95% CI	*P* value*	HR	Lower 95% CI	Upper 95% CI	*P* value*
Age at CAR T^b^	0.990	0.977	1.003	0.1				
Sex (male)	1.19	0.85	1.65	0.3				
CAR T construct (axi-cel)	1.47	0.87	2.50	0.2				
ECOG ≥ 1	0.72	0.52	0.99	0.05	0.76	0.54	1.08	0.1
Lines of therapy prior to CAR T^b^	1.028	0.872	1.084	0.6				
Bridging therapy prior to CAR T	0.66	0.47	0.93	0.02*	0.98	0.67	1.44	0.9
Time from diagnosis to CAR T (months)^b^	1.001	0.999	1.004	0.3				
ANC^b^	0.992	0.948	1.038	0.7				
Hemoglobin^b^	1.17	1.07	1.28	<0.01*	1.029	0.920	1.150	0.6
Platelets^b^	1.003	1.001	1.004	<0.01*	1.003	1.001	1.004	<0.01*
LDH^b^	0.999	0.998	0.999	<0.01*	0.999	0.998	0.999	0.01*
Albumin^b^	2.405	1.647	3.513	<0.01*	1.74	1.09	2.78	0.02*
Prophylactic G-CSF^c^	1.84	1.27	2.67	<0.01*	2.11	1.39	3.20	<0.01*

^*^P < 0.05, indicating statistical significance in univariate or multivariate models, is denoted by asterisks.

^a^Variables with *P* < 0.10 in Cox univariate proportional hazards model were included in the multivariate model.

^b^Continuous hazard ratio (per unit change in regressor).

^c^Prophylactic G-CSF refers to exposure to G-CSF prior to CAR T cell infusion.

A subset of patients developed recurrent neutropenia after initial neutrophil recovery and many of these were treated with G-CSF. The cumulative incidence of later severe neutropenia and/or treatment with G-CSF (since some patients were treated with G-CSF prior to having an ANC < 0.5 × 10^9^/L) after day ten post-CAR T infusion was 24% and 29% at 30- and 90-days, respectively. The cumulative event is shown stratified by prophylactic G-CSF administration in Fig. 1B. Notably, prophylactic G-CSF did not significantly reduce the risk of later severe neutropenia or treatment with G-CSF (Fine-Gray, HR 0.74, 95% CI 0.43–1.25, *P* = 0.26).

### Thrombocytopenia after anti-CD19 CAR T for lymphoma

Fifty patients (25%) developed severe thrombocytopenia (< 20 × 10^9^/L) after CAR T, occurring within median of five days (range, 1–30). Median duration of thrombocytopenia was 24 days (range, 7–120 days). A multivariate logistic regression model was generated, whereby predictors of severe thrombocytopenia included number of prior lines of therapy (OR 1.316 per line, 95% CI 1.023–1.710, *P* = 0.03), platelets (OR 0.992 per 1 × 10^9^/L, 95% CI 0.987–0.997, *P* < 0.01), hemoglobin (OR 0.702 per 1 g/dL, 95% CI 0.533–0.910, *P* = 0.01), and LDH (OR 1.002 per IU/L, 95% CI 1.000–1.004, *P* = 0.04)(Supplemental Table 2). In univariate and multivariate analyses, prophylactic G-CSF did not significantly affect the relative odds of severe thrombocytopenia.

### Antibiotic use and infections after anti-CD19 CAR T for lymphoma

In total, 126 patients (64%) were treated with intravenous (IV) antibiotics after CAR T, chiefly for febrile neutropenia. There was no difference in the proportion of patients treated with IV antibiotics between the prophylactic G-CSF and control groups (64% vs. 63%, respectively, *P* = 0.87). There was a trend towards a shorter duration of IV antibiotic exposure in the prophylactic G-CSF group (6 vs. 8 days, *P* = 0.11). Thirty-seven patients (19%) had documented infections within 30 days after CAR T, including pneumonia (*N* = 11), bacteremia (*N* = 5), and candidemia (*N* = 3). There was no difference in the proportion of infections between the prophylactic G-CSF and control groups (19% vs. 18%, respectively, *P* = 0.84). Within 30 days after CAR T, three patients had fatal infections (two from candidemia, one from bacterial meningitis); two of three occurred in the control group.

### CRS and ICANS after anti-CD19 CAR T for lymphoma

Maximum CRS and ICANS after anti-CD19 CAR T for lymphoma are shown in Table 3. Overall, 84 patients (43%) experienced grade ≥2 CRS, with 11 patients (6%) having grade ≥3 CRS. The frequency of grade ≥2 CRS was higher in the prophylactic G-CSF group (52% vs. 19%, *P* < 0.01), but grade ≥3 CRS was comparable (6% vs. 4%, *P* = 0.52). Moreover, 69 patients (35%) experienced grade ≥2 ICANS, with 37 patients (19%) having grade ≥3 ICANS. The frequency of grade ≥2 ICANS was comparable between the prophylactic G-CSF and control groups (37% vs. 30%, respectively, *P* = 0.41), as well as grade ≥3 ICANS (17% vs. 23%, respectively, *P* = 0.42). Interrogating the control group alone (Supplemental Table 3), there was no significant difference in toxicities between patients who received G-CSF after CAR T (*N* = 42) and G-CSF non-exposed patients (*N* = 15), both for grade ≥2 CRS (21% vs. 13%, respectively, *P* = 0.71) and grade ≥2 ICANS (31% vs. 27%, respectively, *P* = 1.0).

**Table 3:** Maximum CRS and ICANS in lymphoma cohort.

	Prophylactic G-CSF^a^ (*N* = 140)	Control^b^ (*N* = 57)	Overall (*N* = 197)
*CRS grade*^c^			
0	19 (13.6%)	15 (26.3%)	34 (17.3%)
1	48 (34.3%)	31 (54.4%)	79 (40.1%)
2	64 (45.7%)	9 (15.8%)	73 (37.1%)
3	5 (3.6%)	2 (3.5%)	7 (3.6%)
4	4 (2.9%)	0 (0%)	4 (2.0%)
*ICANS grade*^c^			
0	60 (42.9%)	36 (63.2%)	96 (48.7%)
1	28 (20.0%)	4 (7.0%)	32 (16.2%)
2	28 (20.0%)	4 (7.0%)	32 (16.2%)
3	22 (15.7%)	11 (19.3%)	33 (16.8%)
4	2 (1.4%)	2 (3.5%)	4 (2.0%)

^a^Prophylactic G-CSF refers to exposure to G-CSF prior to CAR T cell infusion.

^b^Control group includes patients who either received G-CSF after CAR T cell infusion (*N* = 42) or were not exposed to G-CSF (*N* = 15).

^c^Maximum CRS/ICANS grade per ASTCT consensus criteria.

**Fig. 2:**
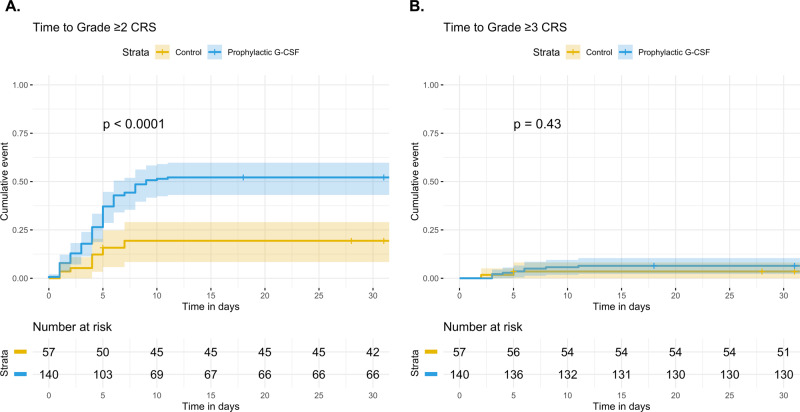
Cumulative incidence of CRS in lymphoma patients treated with anti-CD19 CAR T cells. Time to grade ≥2 CRS (**A**) or grade ≥3 CRS (**B**) were estimated. Patients were stratified by whether they received prophylactic G-CSF (*N* = 140) or not (control, *N* = 57), and compared using the method of Gray. The cumulative incidence of grade ≥2 CRS was significantly greater in the prophylactic G-CSF group (*P* < 0.01). In a multivariate Cox proportional hazards model, administration of prophylactic G-CSF prior to CAR T retained significance for increased risk of grade ≥2 CRS (HR 2.15, 95% CI 1.11–4.18, *P* = 0.02) (see Table 4). 95% confidence intervals are represented by shading.

Time to grade ≥2 and grade ≥3 CRS, stratified by prophylactic G-CSF, are shown in Fig. 2. A multivariate Cox proportional hazards model for time to grade ≥2 CRS was generated. In univariate analysis, CAR T construct, platelets, LDH, and prophylactic G-CSF were significantly associated with increased risk of grade ≥2 CRS (Table 4). In a multivariate analysis that included prophylactic G-CSF, CAR T construct, bridging therapy, ANC, platelets, and LDH, both CAR T construct (axicabtagene ciloleucel, HR 3.48, 95% CI 1.12–10.84, *P* = 0.03) and prophylactic G-CSF exposure retained significance for an increased risk of grade ≥2 CRS (HR 2.15, 95% CI 1.11–4.18, *P* = 0.02). The subgroup of 166 patients treated with axicabtagene ciloleucel were also analyzed separately. Again, prophylactic G-CSF was associated with grade ≥2 CRS in a multivariate analysis (HR 2.00, 95% CI 1.03–3.90, *P* = 0.04)(Supplemental Fig. 1A, Supplemental Table 4).

**Table 4:** Cox proportional hazards model for Grade ≥2 CRS in lymphoma cohort.

Variable	Univariate analysis^a^				Multivariate analysis			
	HR	Lower 95% CI	Upper 95% CI	*P* value*	HR	Lower 95% CI	Upper 95% CI	*P* value*
Age at CAR T^b^	1.002	0.986	1.018	0.8				
Sex (male)	0.76	0.49	1.16	0.2				
CAR T construct (axi-cel)	4.67	1.71	12.75	<0.01*	3.48	1.12	10.84	0.03*
ECOG ≥ 1	1.40	0.91	2.15	0.1				
Lines of therapy prior to CAR T^b^	0.908	0.780	1.056	0.2				
Bridging therapy prior to CAR T	0.64	0.41	1.03	0.06	0.75	0.45	1.25	0.3
Time from diagnosis to CAR T (months)^b^	0.999	0.995	1.004	0.7				
ANC^b^	1.049	0.992	1.109	0.09	1.017	0.955	1.083	0.6
Hemoglobin^b^	0.945	0.845	1.058	0.3				
Platelets^b^	1.003	1.000	1.005	0.03*	1.002	1.000	1.004	0.04*
LDH^b^	1.001	1.000	1.002	0.01*	1.002	1.001	1.002	<0.01*
Albumin^b^	0.709	0.462	1.089	0.1				
Prophylactic G-CSF^c^	3.31	1.75	6.24	<0.01*	2.15	1.11	4.18	0.02*

*P < 0.05, indicating statistical significance in univariate or multivariate models, is denoted by asterisks.

^a^Variables with *P* < 0.10 in Cox univariate proportional hazards model were included in the multivariate model.

^b^Continuous hazard ratio (per unit change in regressor).

^c^Prophylactic G-CSF refers to exposure to G-CSF prior to CAR T cell infusion.

**Fig. 3:**
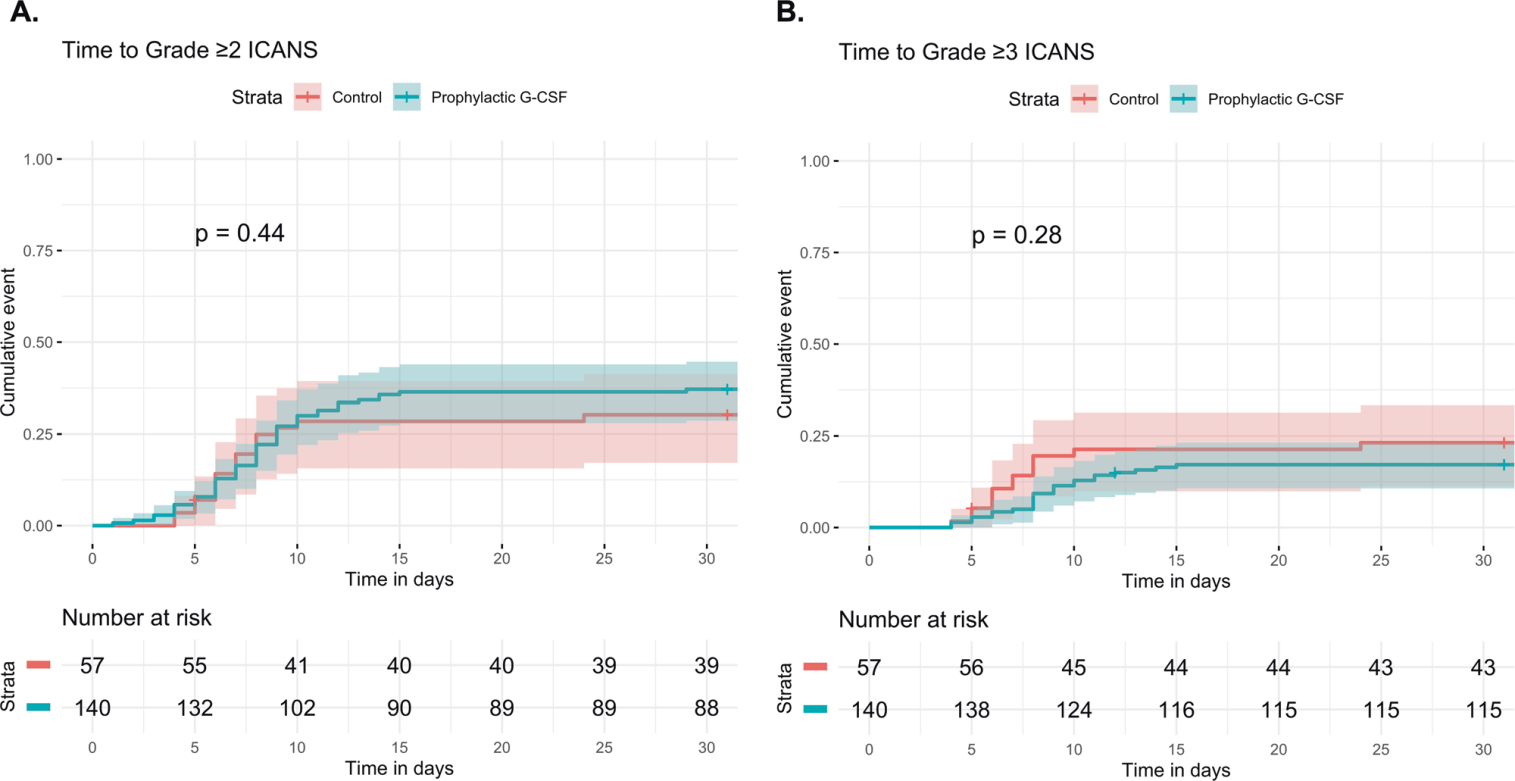
Cumulative incidence of ICANS in lymphoma patients treated with anti-CD19 CAR T cells. Time to grade ≥2 ICANS (**A**) and grade ≥3 ICANS (**B**) were estimated. Patients were stratified by whether they received prophylactic G-CSF (*N* = 140) or not (control, *N* = 57), and compared using the method of Gray. There was no significant difference in the cumulative incidence of grade ≥2 and grade 3 ICANS between the prophylactic G-CSF and control groups, including in a multivariate analysis (see Table 5). 95% confidence intervals are represented by shading.

Time to grade ≥2 and grade ≥3 ICANS, stratified by prophylactic G-CSF, are shown in Fig. 3. Similarly, a Cox proportional hazards model for time to grade ≥2 ICANS was generated. In a multivariate analysis that included prophylactic G-CSF as well as CAR T construct, performance status, hemoglobin, LDH and albumin, only CAR T construct (axicabtagene ciloleucel) retained significance for predicting risk of grade ≥2 ICANS (HR 3.15, 95% CI 1.17–8.47, *P* = 0.02) (Table 5). Prophylactic G-CSF was not associated grade ≥2 ICANS in the multivariate analysis (HR 1.04, 95% CI 0.59–1.84, *P* = 0.9). The findings were similar when the 166 patients treated with axicabtagene ciloleucel were analyzed separately (Supplemental Fig. 1B, Supplemental Table 5).

**Table 5:** Cox proportional hazards model for Grade ≥2 ICANS in lymphoma cohort.

Variable	Univariate analysis^a^				Multivariate analysis			
	HR	Lower 95% CI	Upper 95% CI	*P* value*	HR	Lower 95% CI	Upper 95% CI	*P* value*
Age at CAR T^b^	1.006	0.988	1.024	0.5				
Sex (male)	0.81	0.50	1.31	0.4				
CAR T construct (axi-cel)	2.59	1.04	6.44	0.04*	3.15	1.17	8.47	0.02*
ECOG ≥ 1	1.86	1.14	3.04	0.01*	1.45	0.87	2.41	0.2
Lines of therapy prior to CAR T^b^	1.035	0.892	1.202	0.6				
Bridging therapy prior to CAR T	0.96	0.59	1.57	0.9				
Time from diagnosis to CAR T (months)^b^	0.997	0.991	1.002	0.2				
ANC^b^	1.052	0.987	1.121	0.1				
Hemoglobin^b^	0.781	0.685	0.891	<0.01*	0.897	0.764	1.054	0.2
Platelets^b^	1.000	0.997	1.002	0.7				
LDH^b^	1.001	1.001	1.002	<0.01*	1.001	1.000	1.002	0.08
Albumin^b^	0.388	0.256	0.588	<0.01*	0.673	0.395	1.146	0.1
Prophylactic G-CSF^c^	1.23	0.71	2.13	0.5	1.04	0.59	1.84	0.9

^*^P < 0.05, indicating statistical significance in univariate or multivariate models, is denoted by asterisks.

^a^Variables with *P* < 0.10 in Cox univariate proportional hazards model were included in the multivariate model. The variable of interest, prophylactic G-CSF, was included in the model to study its effect on the development of Grade ≥2 ICANS.

^b^Continuous hazard ratio (per unit change in regressor).

^c^Prophylactic G-CSF refers to exposure to G-CSF prior to CAR T cell infusion.

### Treatment of CRS and ICANS after anti-CD19 CAR T for lymphoma

For CRS and ICANS treatment, 100 patients (51%) received tocilizumab, 91 (46%) received corticosteroids, and seven (4%) received anakinra. In keeping with the documented increased severity of CRS, more patients in the prophylactic G-CSF group received tocilizumab (59% vs. 30%, *P* < 0.01). There was a non-significant trend toward increased corticosteroid administration in the prophylactic G-CSF group as well (50% vs. 37%, respectively, *P* = 0.11).

Twenty-three patients (12%) were admitted to intensive care units (ICU) after CAR T, mostly in the setting of severe CRS and/or ICANS. There was no significant difference in proportion of patients admitted to ICU between the prophylactic G-CSF and control groups (10% vs. 16%, respectively, *P* = 0.33). The duration of hospitalization from CAR T infusion was significantly longer in the prophylactic G-CSF group (median 14 vs. 11 days, *P* = 0.02). However, the rate of re-admission to hospital within 30 days after CAR T infusion was not significantly different between the two groups (10% vs. 16%, respectively, *P* = 0.33).

### Effect of G-CSF initiation in patients with low-grade CRS after anti-CD19 CAR T for lymphoma

**Fig. 4:**
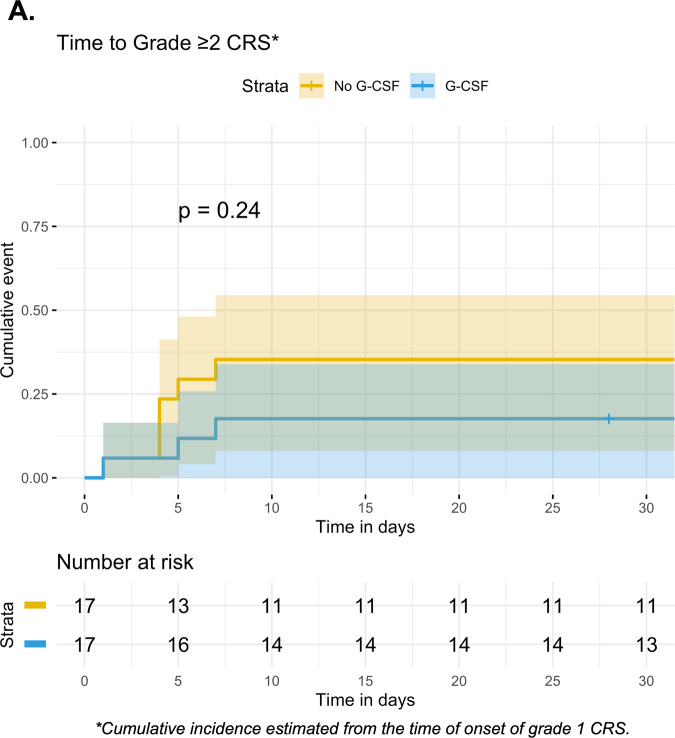
Effect of later G-CSF initiation on CRS severity in G-CSF naïve patients treated with anti-CD19 CAR T cells for lymphoma. **A** Among 34 lymphoma patients with grade ≥1 CRS who had not previously received G-CSF, the cumulative incidence of grade ≥2 CRS was estimated from the time of onset of grade 1 CRS, stratified by whether patients received G-CSF within 7 days after grade 1 CRS onset (*N* = 17) or not (*N* = 17). 95% confidence intervals are represented by shading.

We investigated whether initiating G-CSF worsens the severity of CRS in patients who already had low-grade toxicity. As such, the prophylactic G-CSF group (*N* = 140) from the primary analysis was excluded. Of the remaining 57 G-CSF naïve patients, 42 (74%) went on to experience grade ≥1 CRS. We excluded eight of 42 patients who received G-CSF prior to the onset of grade 1 CRS. Of the remaining 34 eligible patients, 17 (50%) were treated with G-CSF within seven days after the onset of grade 1 CRS, whereas 17 (50%) were not exposed to G-CSF. From the onset of grade 1 CRS, there was no signal of significantly worsened CRS after G-CSF initiation (*P* = 0.24) (Fig. 4A). Similarly, we examined whether initiating G-CSF worsens the severity of low-grade ICANS. However, of the 57 G-CSF naïve patients, just 21 (37%) experienced grade ≥1 ICANS. As such, the number of patients was too few for an analogous investigation of whether later initiation of G-CSF worsens the severity of ICANS.

### Treatment response and survival after anti-CD19 CAR T for lymphoma

For the lymphoma cohort, the overall response rate was 82%, with 134 (68%) complete responses (CR) and 28 (14%) partial responses. Thirty patients (15%) had either stable disease or progression, and five patients (3%) died prior to response assessment. The CR rate was similar between the prophylactic G-CSF and control groups (67% vs. 60%, respectively, *P* = 0.40). For the entire cohort of 197 patients, 2-year PFS and OS were 43.9% (95% CI 37.1–51.9%) and 63.5% (95% CI 56.6–71.2%), respectively (Supplemental Fig. 2). There was no significant difference in survival when comparing prophylactic G-CSF and control groups: 2-year PFS 45.1% vs. 41.0%, respectively (*P* = 0.25); 2-year OS 65.0% vs. 59.6%, respectively, (*P* = 0.18). Overall, seven patients (4%) developed therapy-related myelodysplastic syndrome or acute myeloid leukemia after CAR T, within a median of 18.5 months (range, 5.5–27.3). These patients received significantly more lines of therapy prior to CAR T (5 vs. 3 lines, *P* = 0.04); six received prophylactic G-CSF prior to CAR T.

### Characteristics of patients treated with anti-BCMA CAR T for multiple myeloma

We identified a second cohort of 47 patients with relapsed/refractory multiple myeloma who were treated with anti-BCMA CAR T cells. Unlike the lymphoma cohort, none of these patients received prophylactic G-CSF prior to CAR T per trial protocols. However, 45 of 47 patients (96%) were treated with G-CSF after CAR T per investigator discretion, starting a median of two days after CAR T cell infusion (range, 0–23), for a median of eight doses (range, 1–30). Since we were unable to stratify based on prophylactic G-CSF exposure, we separated patients at the median, i.e. whether they received early G-CSF within two days after CAR T (hereafter referred to as the ‘early G-CSF group’) or not (either G-CSF ≥ 3 days after CAR T or no exposure, hereafter referred to as the ‘control group’). There were no major differences in baseline characteristics between the two groups except that the pre-lymphodepletion platelet count was lower in the early G-CSF group (91 vs. 169 ×10^9^/L, *P* < 0.01) (Table 6).

**Table 6:** Baseline characteristics in multiple myeloma cohort.

	Early G-CSF* (*N* = 24)	Control^a^ (*N* = 23)	Total (*N* = 47)
*Age at CAR T*			
Median [Min, Max]	63 [46, 77]	64 [41, 76]	63 [41, 77]
*Sex*			
Female	7 (29.2%)	9 (39.1%)	16 (34.0%)
*Disease type*			
IgG	14 (58.3%)	9 (39.1%)	23 (48.9%)
IgA	5 (20.8%)	6 (26.1%)	11 (23.4%)
Light chain	5 (20.8%)	7 (30.4%)	12 (25.5%)
Other	0 (0%)	1 (4.3%)	1 (2.1%)
*Number of prior lines of therapy*			
1–3	6 (25.0%)	5 (21.7%)	11 (23.4%)
4–6	10 (41.7%)	13 (56.5%)	23 (48.9%)
≥7	8 (33.3%)	5 (21.7%)	13 (27.7%)
*Prior autologous hematopoietic cell transplant*			
Yes	13 (54.2%)	14 (60.9%)	27 (57.4%)
*ECOG performance status at CAR T*			
0	13 (54.2%)	14 (60.9%)	27 (57.4%)
1	9 (37.5%)	9 (39.1%)	18 (38.3%)
≥2	2 (8.3%)	0 (0%)	2 (4.3%)
*Time from diagnosis to CAR T (months)*			
Median [Min, Max]	70 [7, 149]	68 [8, 130]	70 [7, 149]
*White blood cell count (*×*10*^*9*^*/L)*^b^			
Median [Min, Max]	3.24 [1.82, 7.31]	3.63 [1.32, 12.1]	3.40 [1.32, 12.1]
*Absolute neutrophil count (*×*10*^*9*^*/L)*^b^			
Median [Min, Max]	2.09 [0.92, 4.82]	2.23 [0.90, 9.82]	2.18 [0.90, 9.82]
*Hemoglobin (g/dL)*^b^			
Median [Min, Max]	9.6 [6.9, 13.3]	9.9 [5.7, 12.8]	9.7 [5.7, 13.3]
*Platelet Count (*×*10*^*9*^*/L)*^b^			
Median [Min, Max]	91 [25, 271]	169 [52, 381]	122 [25, 381]

^*^Early G-CSF group received G-CSF within ≤2 days after CAR T.

^a^Control group either received G-CSF ≥ 3 days after CAR T (*N* = 21) or were not exposed to G-CSF (*N* = 2).

^b^Most recent values measured immediately prior to initiation of lymphodepletion chemotherapy.

### Neutropenia and infections after anti-BCMA CAR T for multiple myeloma

Thirty-six of 47 (77%) patients developed severe neutropenia, which was similar between the early G-CSF and control groups (83% vs. 70%, respectively, *P* = 0.32). The overall median duration of neutropenia was 5 days (range, 1–35), and was longer in the early G-CSF group compared to the control group (6 vs. 3 days, *P* = 0.01). Thirty-nine of 47 patients (83%) were treated with IV antibiotics. The overall median duration of IV antibiotic treatment was 8 days (range, 2–32), but was significantly shorter in the early G-CSF group (median 6 vs. 10 days, *P* < 0.01). One severe infection (bacteremia) occurred in each group.

### CRS and ICANS after anti-BCMA CAR T for multiple myeloma

**Fig. 5:**
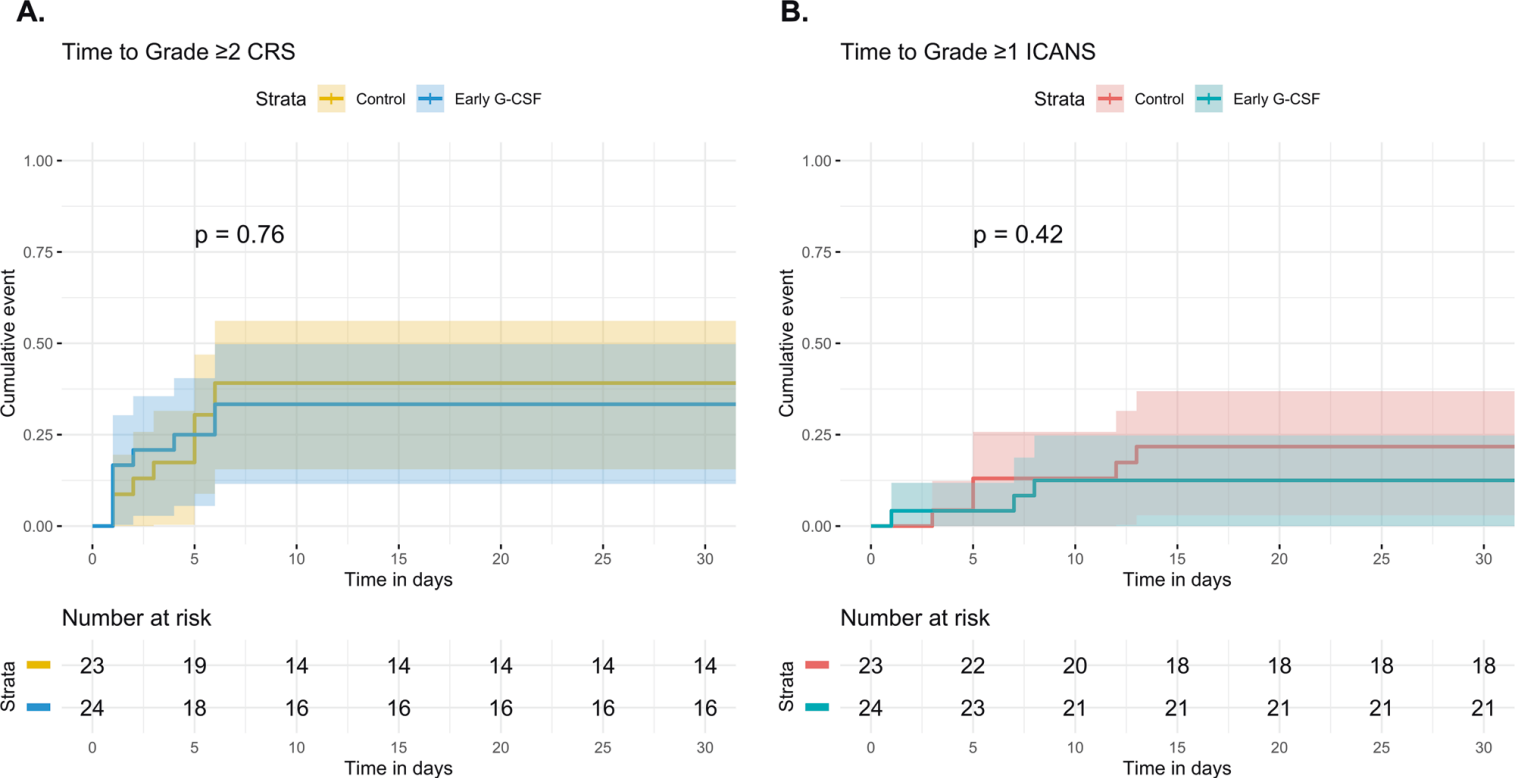
Cumulative incidence of CRS and ICANS in multiple myeloma patients treated with anti-BCMA CAR T cells. Time to grade ≥2 CRS (**A**) and grade ≥1 ICANS (**B**) were estimated and compared by method of Gray, stratified by early G-CSF administration. The early G-CSF group (*N* = 24) received G-CSF within ≤2 days after CAR T, whereas the control group (*N* = 23) either received G-CSF ≥ 3 days after CAR T or were not exposed to G-CSF. 95% confidence intervals are represented by shading.

Overall, grade ≥2 CRS occurred in 17 (36%) of patients, with only one case of grade 3 CRS (Table 7). Grade ≥1 ICANS occurred in eight (17%) patients, with three cases of grade ≥3 ICANS. Time to grade ≥2 CRS and grade ≥1 ICANS were not significantly different between early G-CSF and control groups (*P* = 0.76 and *P* = 0.42, respectively) (Fig. 5). Overall, 24 (51%) of patients received tocilizumab; utilization did not differ between the early G-CSF and control groups (46% vs. 57%, respectively, *P* = 0.56). Thirteen patients (28%) were treated with corticosteroids, which also did not differ (21% vs. 35%, respectively, *P* = 0.34). One patient in each group was admitted to an ICU for CRS/ICANS.

**Table 7:** Maximum CRS and ICANS in multiple myeloma cohort.

	Early G-CSF* (*N* = 24)	Control^a^ (*N* = 23)	Overall (*N* = 47)
*CRS grade*^b^			
0	4 (16.7%)	3 (13.0%)	7 (14.9%)
1	12 (50.0%)	11 (47.8%)	23 (48.9%)
2	8 (33.3%)	8 (34.8%)	16 (34.0%)
3	0 (0%)	1 (4.3%)	1 (2.1%)
*ICANS grade*^b^			
0	21 (87.5%)	18 (78.3%)	39 (83.0%)
1	1 (4.2%)	3 (13.0%)	4 (8.5%)
2	1 (4.2%)	0 (0%)	1 (2.1%)
3	0 (0%)	2 (8.7%)	2 (4.3%)
4	1 (4.2%)	0 (0%)	1 (2.1%)

^*^Early G-CSF group received G-CSF within ≤2 days after CAR T.

^a^Control group either received G-CSF ≥ 3 days after CAR T (*N* = 21) or were not exposed to G-CSF (*N* = 2).

^b^Maximum CRS/ICANS grade per ASTCT consensus criteria.

### Treatment response and survival after anti-BCMA CAR T for multiple myeloma

For the multiple myeloma cohort, the median follow-up time was 20.5 months (95% CI 14.2–25.8). The overall response rate after CAR T was 89%, including 32 (68%) CR. The CR rate was similar between the early G-CSF and control groups (71% vs. 65%, *P* = 0.76). The median PFS was 9.9 months (95% CI 7.4–21.2), and median OS was 23.2 months (95% CI 16.3-not reached)(Supplemental Fig. 3). Survival was not stratified by G-CSF treatment group given the limited number of patients.

## Discussion

In this study, we sought to investigate the effects of G-CSF exposure on CRS, ICANS, hematologic toxicities and infections after CAR T. For the primary analysis, we interrogated the outcomes of 197 lymphoma patients treated at our centers with commercial anti-CD19 CAR T cells. Several recent reports demonstrated that severe CRS increases the risk of hematologic toxicities including prolonged neutropenia [10, 12, 13]. As such, to avoid confounding by indication, we stratified patients based on whether they received G-CSF immediately prior to infusion of CAR T cells, i.e. prophylactic G-CSF. The majority of the 140 patients in the prophylactic G-CSF group were treated with pegylated G-CSF two days prior to CAR T, whereas most of the 57 patients in the control group ultimately received G-CSF starting a median of six days after CAR T to treat neutropenia. As such, the control group largely does not reflect G-CSF naïve patients, but rather later G-CSF initiation after CAR T cell infusion.

Interestingly, after adjusting for multiple baseline variables, including surrogate markers of hematopoietic reserve, disease burden, and CAR T construct type, we found that prophylactic G-CSF exposure (i.e., primarily with pegylated G-CSF) prior to anti-CD19 CAR T was associated with a significantly increased risk of grade ≥2 CRS. However, we did not identify an association between prophylactic G-CSF and severe ICANS. In spite of these findings, prophylactic G-CSF exposure was associated with a small but significantly faster time to neutrophil recovery (3 vs. 4 days); however, the clinical significance of this finding is uncertain as the rate of infections was not significantly different between the prophylactic G-CSF and control groups. There was a trend toward a shorter duration of IV antibiotic exposure with prophylactic G-CSF, but also a longer length of stay in the hospital. Of note, prophylactic G-CSF did not translate to a decreased risk of recurrent severe neutropenia later, reflecting the recognized risk of prolonged cytopenias after CAR T in a substantial proportion of patients [10, 11, 13]. Finally, there was no association between prophylactic G-CSF and treatment response, PFS or OS in lymphoma.

We also interrogated a smaller cohort of 47 multiple myeloma patients treated with anti-BCMA CAR T cells. None of these patients received prophylactic G-CSF, but the majority were treated with G-CSF shortly after CAR T. As such, we analyzed the effect of early G-CSF (within 2 days after CAR T) vs. control (G-CSF ≥ 3 days after CAR T or no exposure). We did not identify a difference in CRS or ICANS between the two groups. There was a longer duration of neutropenia in the early G-CSF group, which is counterintuitive, but may reflect confounding by indication, i.e., patients with more severe hematologic toxicity necessitated earlier G-CSF treatment. While this is a limitation of our anti-BCMA CAR T analysis, it highlights a strength of our anti-CD19 CAR T analysis, given the latter was based on prophylactic G-CSF exposure prior to CAR T.

Several retrospective studies have explored the effects of G-CSF on outcomes after CAR T in lymphoma. A group from Mayo Clinic compared 35 patients who received G-CSF after CAR T to 35 patients who did not—with no signal of increased severity of CRS or ICANS, and shorter duration of neutropenia with post-CAR T G-CSF [27]. Another study compared 42 patients who received G-CSF five days after CAR T to 28 G-CSF non-exposed patients, and found no difference in CRS or ICANS [25]. Liévin et al. also compared 33 patients given G-CSF two days after CAR T with 89 patients either given G-CSF five days after CAR T or not at all; there was no difference in toxicities between the two strategies [26]. Since the patients in the Liévin et al. study had G-CSF use similar to our study’s control group, it could be a benchmark for comparison: Our control group had a similar rate of CRS (74% vs. 73%, respectively), but a higher rate of ICANS (37% vs. 26%, respectively) [26]. The main limitation of the aforementioned studies was the lower number of patients and lack of multivariate analyses to address baseline differences between the cohorts. Further, the separation of patients by patterns of G-CSF use after CAR T could have resulted in confounding by indication, as noted previously.

Indeed, a strength of our analysis is that we stratified based on G-CSF exposure prior to CAR T, which, to the best of our knowledge, is the first study to address this question. Another strength is that we re-graded toxicities based on ASTCT consensus recommendations [35]. We also made use of time-to-event multivariate analyses that included known variables that associated with severity of both CRS and ICANS [1, 21, 39]. Further, no prior studies have explored the effect of G-CSF on outcomes after anti-BCMA CAR T in multiple myeloma. The main weakness of our study is the retrospective design. In the lymphoma cohort, although the G-CSF and control groups were comparable at baseline, not all differences can be adjusted for, and residual confounding remains a challenge. Also given the time range of the study, it is possible that some of the CRS or ICANS management strategies would now be incongruent with current guidelines.

Future clinical trials should explore the optimal safe use of G-CSF in patients treated with CAR T. Until then, our results suggest a strategy where G-CSF is used after CAR T as a treatment for severe neutropenia as opposed to prophylaxis prior to CAR T could be associated with less severe CRS. However, this strategy must be balanced by potential risks associated with a longer period of early neutropenia. Though limited by small numbers, we did not find that later initiation of G-CSF in patients who already had low-grade CRS significantly exacerbated toxicity—further evidence that a neutropenia-driven G-CSF treatment approach could be feasible. Perhaps by identifying patients with risk factors for prolonged severe neutropenia (i.e. with multivariate models described herein or elsewhere) [12], methods to preemptively mitigate hematologic toxicity with earlier G-CSF administration or more aggressive CRS management could be advantageous, but these hypotheses require prospective investigation.

Mechanistically, we hypothesize that G-CSF given prior to CAR T primes myeloid cells at the critical juncture before CAR T in vivo expansion, resulting in intensified cytokine cross-talk and thus increased severity of CRS, but this theory requires elucidation with translational studies [17, 19]. The biologic mechanisms governing long-term hematologic toxicities after CAR T also require further unraveling to devise potential therapeutic interventions. Discovery of novel methods to prevent or attenuate CRS and ICANS, such as with prophylactic anakinra, remain a clinical need [40, 41].

## Supplementary information

Supplemental Material

## Data Availability

The data generated and analyzed during the current study are not publicly available due to institutional policies but may be available from the corresponding author on reasonable request.
