# Supplementary material for: Effect of granulocyte colony-stimulating factor on toxicities after CAR T cell therapy for lymphoma and myeloma
Source: Blood Cancer J. 2022 Nov 1;12(10):146. doi: 10.1038/s41408-022-00741-2 (PMC9622902; doi:10.1038/s41408-022-00741-2)
Supplement: Supplementary file 1 — Supplemental Material [file 41408_2022_741_MOESM1_ESM.pdf]

SUPPLEMENTAL MATERIAL

Effect of Granulocyte Colony-Stimulating Factor on Toxicities after CAR T cell Therapy for Lymphoma and Myeloma

Miller, K.C., et al.

Supplemental Table 1. Page 2
Logistic Regression Model for Severe Neutropenia in Lymphoma Cohort

Supplemental Table 2. Page 3
Logistic Regression Model for Severe Thrombocytopenia in Lymphoma Cohort

Supplemental Table 3. Page 4
Maximum CRS and ICANS in Lymphoma Cohort by G-CSF exposure

Supplemental Table 4. Page 5
Cox Proportional Hazards Model for Grade ≥2 CRS in Lymphoma Patients treated with axicabtagene ciloleucel

Supplemental Table 5. Page 6
Cox Proportional Hazards Model for Grade ≥2 ICANS in Lymphoma Patients treated with axicabtagene ciloleucel

Supplemental Figure 1. Page 7
Cumulative incidence of CRS and ICANS for the subset of lymphoma patients treated with axicabtagene ciloleucel

Supplemental Figure 2. Page 8
PFS and OS for patients with lymphoma who received anti-CD19 CAR T cells.

Supplemental Figure 3. Page 9
PFS and OS for patients with multiple myeloma who received anti-BCMA CAR T cells.

Supplemental Figure Legends. Page 10

Supplemental Table 1. Logistic Regression Model for Severe Neutropenia in Lymphoma Cohort

	Univariate Analysis§				Multivariate Analysis			
Variable	**OR**	*Lower 95% CI*	*Upper 95% CI*	*P* value*	**OR**	*Lower 95% CI*	*Upper 95% CI*	*P* value*
Age at CAR T†	**1.005**	0.981	1.029	0.7				
Sex (male)	**0.71**	0.35	1.41	0.3				
CAR T construct (axi-cel)	**2.45**	1.06	5.50	0.03*	**5.96**	1.90	21.20	<0.01*
ECOG ≥1	**2.20**	1.12	4.41	0.02*	**1.67**	0.79	3.59	0.2
Lines of therapy prior to CAR T†	**1.280**	1.009	1.671	0.05	**1.001**	0.999	1.004	0.07
Bridging therapy prior to CAR T	**1.44**	0.73	2.95	0.3				
Time from Diagnosis to CAR T (months)†	**1.001**	0.995	1.007	0.9				
ANC†	**1.083**	0.965	1.242	0.2				
Hemoglobin†	**0.810**	0.668	0.972	0.03*	**0.906**	0.720	1.137	0.4
Platelets†	**0.998**	0.995	1.002	0.4				
LDH†	**1.002**	1.000	1.004	0.06	**1.001**	0.999	1.004	0.3
Albumin†	**0.408**	0.171	0.900	0.03*	**0.656**	0.233	1.797	0.4
Prophylactic G-CSF‡	**0.83**	0.38	1.72	0.6	**0.33**	0.10	0.93	0.05

*P<0.05, indicating statistical significance in univariate or multivariate models, is denoted by asterisks.
†Continuous hazard ratio (per unit change in regressor).
‡Prophylactic G-CSF refers to exposure to G-CSF prior to CAR T cell infusion.
§Variables with P<0.10 in univariate logistic regression model were included in the multivariate model (represented by grey shading). The variable of interest, prophylactic G-CSF, was included in the multivariate model to study its effect on the development of severe neutropenia.

Supplemental Table 2. Logistic Regression Model for Severe Thrombocytopenia in Lymphoma Cohort

	Univariate Analysis§				Multivariate Analysis			
Variable	**OR**	*Lower 95% CI*	*Upper 95% CI*	*P* value*	**OR**	*Lower 95% CI*	*Upper 95% CI*	*P* value*
Age at CAR T†	**0.998**	0.975	1.023	0.9				
Sex (male)	**0.74**	0.39	1.43	0.4				
CAR T construct (axi-cel)	**1.50**	0.61	4.26	0.4				
ECOG ≥1	**2.79**	1.42	5.67	<0.01*	**1.66**	0.72	3.91	0.2
Lines of therapy prior to CAR T†	**1.343**	1.095	1.659	0.01*	**1.316**	1.023	1.710	0.03*
Bridging therapy prior to CAR T	**2.55**	1.33	4.94	0.01*	**1.70**	0.69	4.19	0.3
Time from Diagnosis to CAR T (months)†	**0.997**	0.990	1.003	0.4				
ANC†	**1.074**	0.972	1.187	0.2				
Hemoglobin†	**0.556**	0.443	0.683	<0.01*	**0.702**	0.533	0.910	0.01*
Platelets†	**0.989**	0.984	0.994	<0.01*	**0.992**	0.987	0.997	<0.01*
LDH†	**1.003**	1.002	1.005	<0.01*	**1.002**	1.000	1.004	0.04*
Albumin†	**0.172**	0.074	0.367	<0.01*	**0.427**	0.140	1.216	0.1
Prophylactic G-CSF‡	**0.82**	0.41	1.67	0.6	**1.02**	0.41	2.66	1.0

**P*<0.05, indicating statistical significance in univariate or multivariate models, is denoted by asterisks.
†Continuous hazard ratio (per unit change in regressor).
‡Prophylactic G-CSF refers to exposure to G-CSF prior to CAR T cell infusion.
§Variables with *P*<0.10 in univariate logistic regression model were included in the multivariate model (represented by grey shading). The variable of interest, prophylactic G-CSF, was included in the model to study its effect on the development of severe thrombocytopenia.

Supplemental Table 3. Maximum CRS and ICANS in Lymphoma Cohort by G-CSF Exposure

	Prophylactic G-CSF* (N=140)	G-CSF after CAR T† (N=42)	G-CSF non-exposed† (N=15)	Overall (N=197)
**CRS grade**				
0	19 (13.6%)	10 (23.8%)	5 (33.3%)	34 (17.3%)
1	48 (34.3%)	23 (54.8%)	8 (53.3%)	79 (40.1%)
2	64 (45.7%)	7 (16.7%)	2 (13.3%)	73 (37.1%)
3	5 (3.6%)	2 (4.8%)	0 (0%)	7 (3.6%)
4	4 (2.9%)	0 (0%)	0 (0%)	4 (2.0%)
**ICANS grade**				
0	60 (42.9%)	26 (61.9%)	10 (66.7%)	96 (48.7%)
1	28 (20.0%)	3 (7.1%)	1 (6.7%)	32 (16.2%)
2	28 (20.0%)	3 (7.1%)	1 (6.7%)	32 (16.2%)
3	22 (15.7%)	8 (19.0%)	3 (20.0%)	33 (16.8%)
4	2 (1.4%)	2 (4.8%)	0 (0%)	4 (2.0%)

*Prophylactic G-CSF refers to exposure to G-CSF prior to CAR T cell infusion.
†Within 30 days after CAR T cell infusion.

Supplemental Table 4. Cox Proportional Hazards Model for Grade ≥2 CRS in Lymphoma Patients treated with axicabtagene ciloleucel

	Univariate Analysis §				***Multivariate Analysis***			
Variable	**HR**	*Lower 95% CI*	*Upper 95% CI*	*P* value*	**HR**	*Lower 95% CI*	*Upper 95% CI*	*P* value*
Age at CAR T†	**1.009**	0.992	1.027	0.3				
Sex (male)	**0.68**	0.44	1.06	0.1				
ECOG ≥1	**1.42**	0.91	2.21	0.1				
Lines of therapy prior to CAR T†	**0.874**	0.746	1.024	0.1				
Bridging therapy prior to CAR T	**0.88**	0.54	1.43	0.6				
Time from Diagnosis to CAR T (months)†	**0.998**	0.994	1.003	0.5				
ANC†	**1.042**	0.983	1.106	0.2				
Hemoglobin†	**0.935**	0.836	1.045	0.2				
Platelets†	**1.002**	0.999	1.004	0.08	**1.002**	0.999	1.004	0.07
LDH†	**1.001**	1.001	1.002	<0.01*	**1.002**	1.001	1.002	<0.01*
Albumin†	**0.728**	0.479	1.108	0.1				
Prophylactic G-CSF‡	**1.90**	0.98	3.69	0.06	**2.00**	1.03	3.90	0.04*

**P*<0.05, indicating statistical significance in univariate or multivariate models, is denoted by asterisks.
†Continuous hazard ratio (per unit change in regressor).
‡Prophylactic G-CSF refers to exposure to G-CSF prior to CAR T cell infusion.
§Variables with *P*<0.10 in Cox univariate proportional hazards model were included in the multivariate model (represented by grey shading).

Supplemental Table 5. Cox Proportional Hazards Model for Grade ≥2 ICANS in Lymphoma Patients treated with axicabtagene ciloleucel

	Univariate Analysis§				*Multivariate Analysis*			
Variable	**HR**	*Lower 95% CI*	*Upper 95% CI*	*P* value*	**HR**	*Lower 95% CI*	*Upper 95% CI*	*P* value*
Age at CAR T†	**1.012**	0.992	1.033	0.2				
Sex (male)	**0.83**	0.50	1.36	0.5				
ECOG ≥1	**2.20**	1.31	3.71	<0.01*	**1.74**	1.02	2.98	0.04*
Lines of therapy prior to CAR T†	**1.007**	0.862	1.176	0.9				
Bridging therapy prior to CAR T	**1.10**	0.66	1.86	0.7				
Time from Diagnosis to CAR T (months)†	**0.996**	0.991	1.002	0.2				
ANC†	**1.058**	0.989	1.131	0.1				
Hemoglobin†	**0.782**	0.684	0.894	<0.01*	**0.942**	0.795	1.117	0.5
Platelets†	**0.999**	0.997	1.002	0.7				
LDH†	**1.002**	1.001	1.003	<0.01*	**1.002**	1.000	1.003	0.01*
Albumin†	**0.377**	0.250	0.567	<0.01*	**0.674**	0.403	1.129	0.1
Prophylactic G-CSF‡	**1.01**	0.54	1.89	1.0	**1.36**	0.71	2.61	0.4

**P*<0.05, indicating statistical significance in univariate or multivariate models, is denoted by asterisks.
†Continuous hazard ratio (per unit change in regressor).
‡Prophylactic G-CSF refers to exposure to G-CSF prior to CAR T cell infusion.
§Variables with *P*<0.10 in Cox univariate proportional hazards model were included in the multivariate model (represented by grey shading). The variable of interest, prophylactic G-CSF, was included in the model to study its effect on the development of Grade ≥2 ICANS.

Supplemental Figure 1.

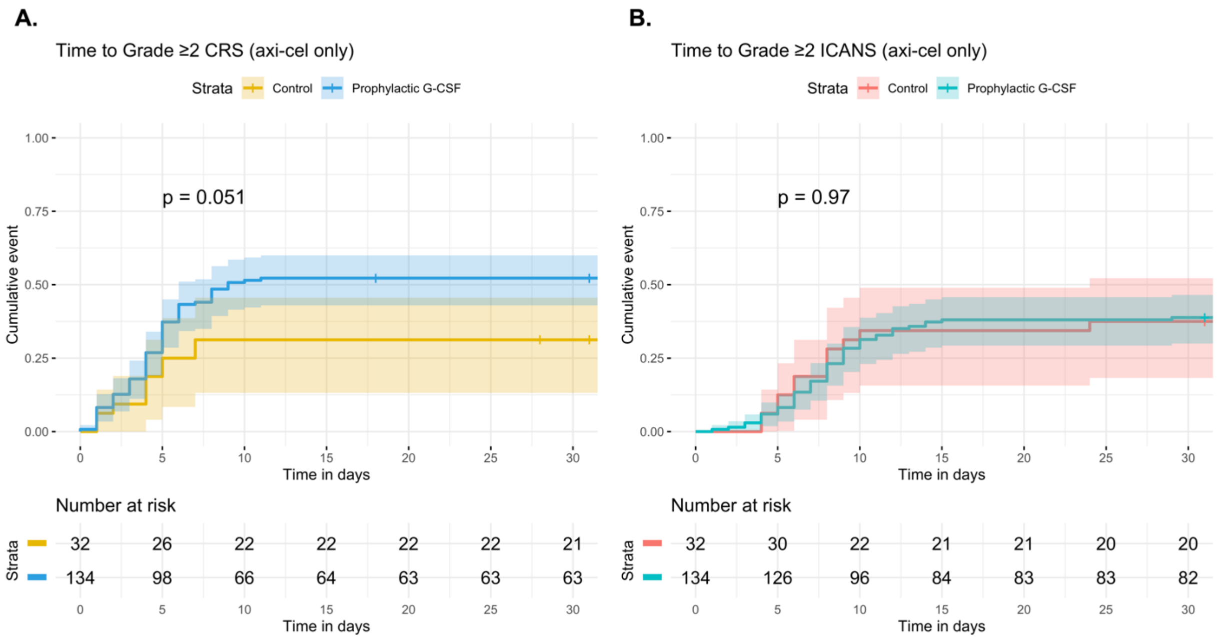

Supplemental Figure 2.

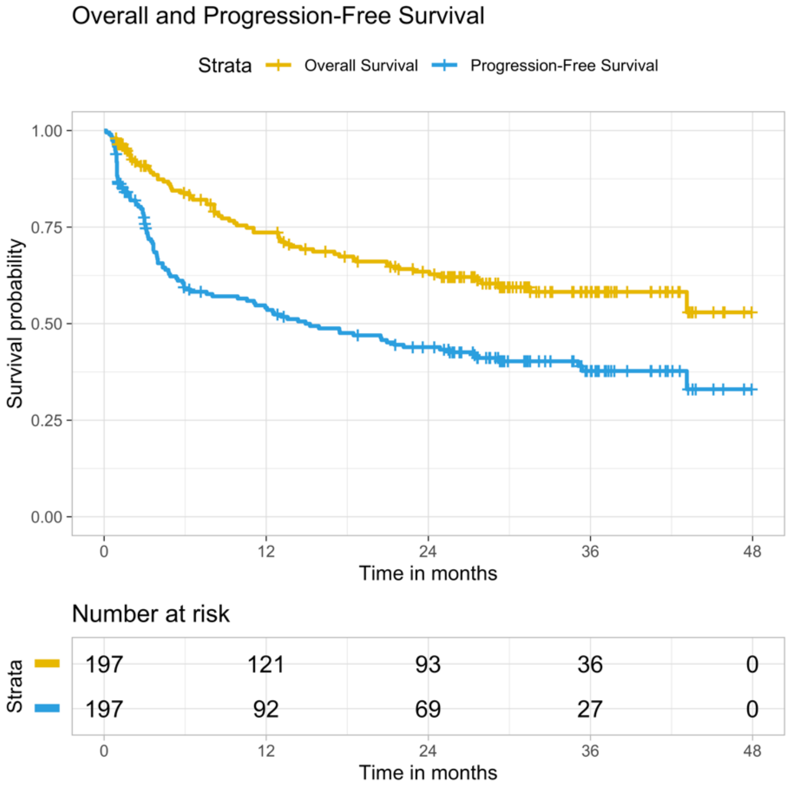

Supplemental Figure 3.

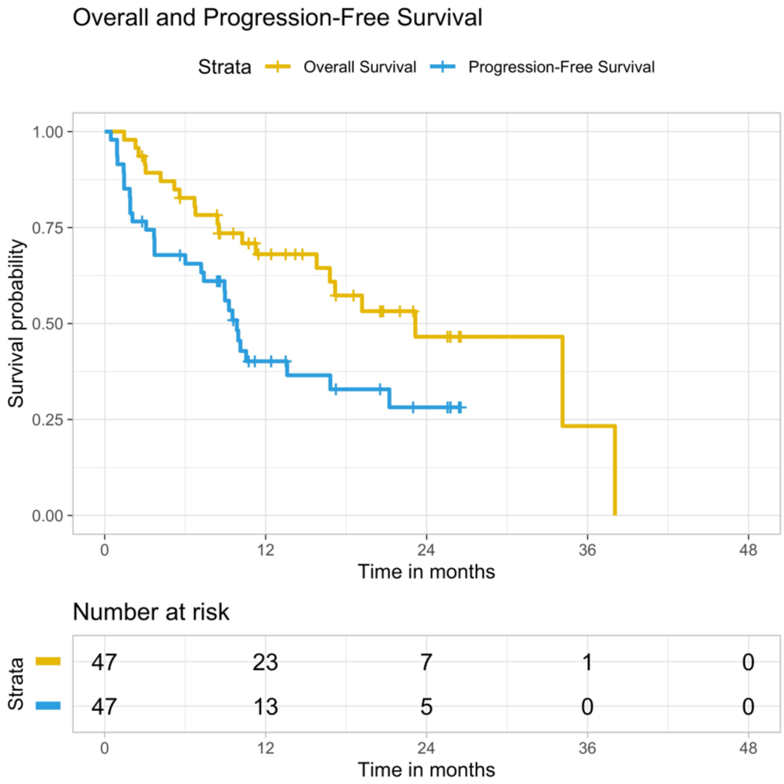

SUPPLEMENTAL FIGURE LEGENDS

Supplemental Figure 1.
Cumulative incidence of CRS and ICANS for the subset of lymphoma patients treated with axicabtagene ciloleucel (N=166). Time to grade ≥2 CRS (**Panel A**) and grade ≥2 ICANS (**Panel B**) were estimated. Patients were stratified by whether they received prophylactic G-CSF (N=134) or not (control, N=32). There was a trend toward a greater cumulative incidence of grade ≥2 CRS in the prophylactic G-CSF group (Gray test, *P*=0.051). However, in a multivariate analysis (see Supplemental Table 4), prophylactic G-CSF was significantly associated with grade ≥2 CRS (HR 2.00, 95% CI 1.03-3.90, *P*=0.04). There was no significant difference in grade ≥2 ICANS between the two groups. 95% confidence intervals are represented by shading.

Supplemental Figure 2.
Progression-free survival (PFS) and overall survival (OS) for patients with lymphoma who received anti-CD19 CAR T cells. 2-year PFS was 43.9% (95% CI 37.1-51.9%). 2-year OS was 63.5% (95% CI 56.6-71.2%).

Supplemental Figure 3.
Progression-free survival (PFS) and overall survival (OS) for patients with multiple myeloma who received anti-BCMA CAR T cells. Median PFS was 9.9 months (95% CI 7.4-21.2). Median OS was 23.2 months (95% CI 16.3-not reached).
